# Clinical Evidence of Wearable-Derived Heart Rate Variability for Detecting Systemic Inflammation: A Systematic Review

**DOI:** 10.3390/diagnostics16040538

**Published:** 2026-02-11

**Authors:** Rukmono Siswishanto, Detty Siti Nurdiati, Irwan Endrayanto Aluicius, Aulia Ichlasul Rezza, Dean Batrisha

**Affiliations:** 1 Department of Obstetrics and Gynecology, Faculty of Medicine, Public Health and Nursing, Universitas Gadjah Mada, Yogyakarta 55281, Indonesia; detty@ugm.ac.id (D.S.N.); aulia.ichlasul.rezza@mail.ugm.ac.id (A.I.R.); deanbatrisha@mail.ugm.ac.id (D.B.); 2 Department of Mathematics, Faculty of Mathematics and Natural Sciences, Universitas Gadjah Mada, Yogyakarta 55281, Indonesia; endrayanto@ugm.ac.id

**Keywords:** heart rate variability, wearable devices, systemic inflammation, SDNN, RMSSD, inflammatory biomarkers

## Abstract

**Background/Objectives**: Wearable devices capable of capturing heart rate variability (HRV) enable continuous assessment of autonomic nervous system function in real-world settings. Because systemic inflammation disrupts autonomic balance through vagal withdrawal and sympathetic activation, HRV has been proposed as a non-invasive digital biomarker of inflammatory activity. Despite the rapid expansion of wearable sensor technologies, the accuracy and consistency for detecting systemic inflammatory states remain unclear. This systematic review aimed to evaluate the clinical relevance of wearable-derived HRV indices in relation to established inflammatory biomarkers. **Methods**: A systematic search of PubMed, Scopus, Web of Science, and the Cochrane Library was conducted through April 2025. Due to methodological heterogeneity, findings were synthesized using the Synthesis Without Meta-analysis (SWiM) framework with vote counting, effect-direction plots, and sign tests. **Results:** Eleven studies involving 2419 participants met the inclusion criteria. Vote counting demonstrated that SDNN showed a predominantly inverse association with CRP, with 83% of comparisons indicating reduced SDNN in the presence of elevated CRP (sign test *p* = 0.031). In contrast, associations between RMSSD and inflammatory cytokines were heterogeneous and largely non-significant. ECG-based wearable devices yielded more consistent associations than photoplethysmography-based devices, while recording duration and population characteristics contributed to variability across studies. **Conclusions**: Wearable-derived HRV, particularly SDNN from ECG-based devices, shows a consistent inverse association with CRP, supporting its role as a non-invasive physiological correlate of systemic inflammation. However, heterogeneity and the lack of diagnostic accuracy metrics limit conclusions regarding clinical utility. At present, wearable HRV should be considered an exploratory or adjunctive biomarker, pending validation in standardized longitudinal studies with formal diagnostic performance assessment.

## 1. Introduction

Inflammation is a fundamental physiological response of the immune system to a wide range of pathological conditions and plays a critical role in the progression of numerous diseases, including autoimmune disorders, metabolic syndrome, cardiovascular disease, and infections. Accurate monitoring of inflammatory status is therefore essential for assessing disease activity, evaluating progression, and guiding therapeutic decision-making. Currently, circulating biomarkers such as C-reactive protein (CRP), interleukins (e.g., IL-6), and serum amyloid A are among the most commonly used indicators of systemic inflammation [1]. However, several of these markers are disease-specific, require invasive sampling, and are measured intermittently, which limits their feasibility for continuous or routine monitoring.

Alterations in autonomic nervous system (ANS) activity have been closely linked to inflammatory processes. Inflammatory states are commonly associated with reduced vagal tone and heightened sympathetic activity, resulting in decreased heart rate variability (HRV) [2]. This interaction is mediated in part through the vagus nerve, which plays a central role in the cholinergic anti-inflammatory reflex by inhibiting the release of pro-inflammatory cytokines. Consequently, diminished vagal activity and thus reduced HRV has been associated with heightened systemic inflammation [3]. HRV, defined as the variation in time intervals between consecutive heartbeats, reflects the integrated function of multiple regulatory systems, particularly the ANS, and has been widely used as an indicator of physiological health and autonomic balance [4,5]. Increased sympathetic activity combined with reduced parasympathetic modulation leads to lower HRV values, a pattern frequently observed during inflammatory responses [4,6]. Previous studies have reported inverse associations between HRV and inflammatory biomarkers, supporting a potential link between autonomic regulation and immune activity [7].

Among the various HRV metrics, two time-domain indices, the standard deviation of normal-to-normal intervals (SDNN) and the root mean square of successive differences (RMSSD), are the most widely used and validated in both clinical and ambulatory settings [4]. SDNN reflects overall heart rate variability and captures combined sympathetic and parasympathetic influences over longer recording periods, showing strong correlations with frequency-domain indices such as ultra-low-frequency (ULF), very-low-frequency (VLF), and low-frequency (LF) power, as well as total power. In contrast, RMSSD primarily reflects short-term parasympathetic (vagal) activity and is commonly used in shorter recordings, correlating with indices such as SD1, pNN50, and high-frequency (HF) power [8,9]. Importantly, both SDNN and RMSSD are relatively robust to signal artifacts and can be derived from electrocardiographic (ECG) and photoplethysmographic (PPG) signals, making them particularly suitable for wearable-based HRV monitoring [9,10].

The increasing availability of wearable technologies has enabled HRV monitoring in real-world conditions outside traditional clinical environments. Wearable devices incorporating ECG or PPG sensors commonly embedded in smartwatches, fitness bands, and portable ECG patches allow continuous HRV data acquisition during daily activities [5,11]. Emerging evidence suggests that wearable-derived HRV may be associated with inflammatory states. For example, studies conducted during the COVID-19 pandemic reported changes in wearable-derived HRV preceding or accompanying positive PCR test results [12,13]. Similar findings have been observed in chronic inflammatory conditions such as inflammatory bowel disease, where consumer-grade wearable devices demonstrated potential for anticipating disease flares [14].

From a diagnostic perspective, systemic inflammation is currently identified using circulating biomarkers that serve as clinical reference standards but are limited by invasiveness, cost, and intermittent sampling. Wearable-derived physiological signals such as HRV offer a potential adjunctive approach for inflammation detection and monitoring. However, before HRV can be incorporated into diagnostic pathways, its clinical accuracy, consistency across devices, and robustness across inflammatory contexts must be critically evaluated [15,16]. The absence of standardized diagnostic thresholds and formal validation of diagnostic performance underscores the need for systematic assessment of existing evidence.

In this review, systemic inflammation was operationalized broadly to encompass clinical inflammatory conditions, biomarker-defined inflammatory states, and experimentally induced inflammatory responses. Included studies defined inflammation using established circulating biomarkers (e.g., CRP and cytokines), clinical diagnoses associated with inflammatory activity, or controlled experimental models such as endotoxemia. These approaches were treated as conceptually related but biologically heterogeneous manifestations of systemic inflammatory activity.

Despite growing interest in wearable HRV monitoring for inflammation detection [5,13], its clinical applicability remains uncertain. To date, no systematic review has comprehensively synthesized evidence comparing wearable-derived HRV indices with established inflammatory biomarkers across diverse clinical and experimental contexts. Therefore, this systematic review aims to evaluate the associations between wearable-derived HRV indices and conventionally used inflammatory biomarkers, in order to assess the consistency and potential clinical relevance of HRV as a non-invasive physiological correlate of systemic inflammatory activity.

## 2. Materials and Methods

This systematic review was conducted in accordance with the Preferred Reporting Items for Systematic Reviews and Meta-Analyses (PRISMA) guidelines (Figure S1). The review protocol was prospectively registered in the International Prospective Register of Systematic Reviews (PROSPERO) under the registration number CRD420251029821.

### 2.1. Data Sources and Search Strategy

A comprehensive literature search was conducted in April 2025 using the following electronic databases: PubMed/MEDLINE, Web of Science, and Scopus, without restrictions on publication year. Ongoing and registered studies were also screened via ClinicalTrials.gov and the Cochrane trial registry. The search strategy incorporated Medical Subject Headings (MeSH) and title/abstract terms using the keywords “heart rate variability”, “inflammation”, and “wearable devices”. Additional records were identified through manual searches using academic search engines and by screening the reference lists of included studies. The full search strategies for each database are provided in Supplementary Table S1.

### 2.2. Eligibility Criteria and Outcomes

Several eligibility criteria were predefined and applied to guide the assessment of retrieved records. Studies were included if they met all of the following criteria: (1) randomized or non-randomized study designs enrolling adult participants aged 18 years or older with diagnosed or suspected systemic inflammation; (2) assessment of heart rate variability (HRV) using wearable or ambulatory monitoring devices, including chest straps, wrist-worn devices, or biosensor patches; (3) reporting of time-domain HRV indices, specifically the standard deviation of normal-to-normal intervals (SDNN) and/or the root mean square of successive differences (RMSSD); (4) comparison of wearable-derived HRV measures with established inflammatory biomarkers, including C-reactive protein (CRP), interleukin-6 (IL-6), and/or tumor necrosis factor-α (TNF-α); and (5) reporting of original data from randomized controlled trials, non-randomized trials, interrupted time-series studies, or before–after study designs. Studies were excluded if they met any of the following criteria: (1) inclusion of exclusively healthy or pediatric populations; (2) use of stationary, non-wearable electrocardiography systems; (3) availability only as abstracts, editorials, letters, case reports, protocols, reviews, books, or conference papers; (4) lack of full-text availability or insufficient data for extraction; or (5) publication in languages other than English or Indonesian. Eligibility assessment was conducted independently by two investigators, with disagreements resolved through discussion and consensus involving a third reviewer.

### 2.3. Study Selection

A two-step study selection process was employed with two authors (AIR & DB) independently screened titles and abstracts of studies to exclude studies that did not meet the inclusion criteria. Consequently, the full texts of the studies were also independently assessed to determine final eligibility for inclusion, while documenting reasons for exclusion. Discrepancies between the reviewers were resolved through discussion with a third reviewer (RS). The screening process was carried out using Rayyan software (https://rayyan.ai, accessed from March 2025 to June 2025).

### 2.4. Data Extraction and Management

The search results were imported and managed using Rayyan, a web-based tool for systematic reviews, and Zotero reference manager 7.0.5. We used Rayyan’s deduplication feature to identify and remove duplicate records, followed by manual verification by the primary investigator. We reported the result of the study selection process in detail via the PRISMA flow diagram. The data extracted includes (1) study details: authors, year of publication, location of origin, sex, study type, population condition, number of participants, and, if available, study intervention; (2) intervention characteristic: recording device, HRV parameters, record time, inflammation biomarkers; (3) main key findings. Discrepancies between the two reviewers were resolved through consultation with the third author. When study data were missing or unclear, the authors of the original studies were contacted for clarification or additional information.

### 2.5. Quality and Risk of Bias Management

Risk of bias was assessed using the NIH Quality Assessment Tool for Observational Cohort and Cross-Sectional Studies and the Cochrane risk-of-bias 2 tool for RCTs. Based on the assessment, studies were grouped as low, high, or unclear.

### 2.6. Data Synthesis and Analysis

A comprehensive systematic review was conducted to evaluate the association between HRV and inflammatory markers across various inflammatory conditions. Due to the heterogeneity of studies, parameters and outcomes, conducting a formal meta-analysis was impossible. Thus, a systematic review was conducted in accordance with the Synthesis Without Meta-analysis (SWiM) guideline [17] as recommended by the Cochrane Foundation. Randomized and observational studies were synthesized together to assess the consistency of associations between wearable-derived HRV and inflammatory biomarkers rather than to infer causal effects, with study design considered descriptively rather than hierarchically weighted. An effect direction plot was used to visually summarize the findings following the Cochrane handbook [18].

In this study, upward arrows represented a positive association between HRV indices and inflammatory markers, indicating that when inflammatory markers were elevated in inflammatory populations, HRV indices also tended to be higher. Downward arrows represented a negative association, reflecting that higher levels of inflammatory markers were accompanied by lower HRV indices. A bidirectional arrow was used when the study reported unclear or conflicting association. This categorization was based on evaluating mean or median differences, correlation coefficients, odds’ ratio, and/or coefficients from linear regression analysis as provided by the papers. To reduce bias and maintain consistency across studies, data from interventional designs were restricted to baseline measurements. Similarly, studies involving only healthy participants were excluded unless inflammatory subgroup data were reported, to ensure that comparisons reflected populations with relevant inflammatory activity. When studies reported multiple inflammatory biomarkers, a single representative HRV biomarker comparison was selected per study for sign-test analysis to preserve statistical independence, with priority given to the primary or most clinically relevant biomarker reported, with CRP prioritized when available.

Subsequently, a nonparametric sign test was conducted to assess the direction of effects across studies. An estimate of proportions of negative effects was also calculated as *p* = u/n, where u = number of negative relationships, and n = number of studies, alongside 95% confidence intervals using Wilson’s confidence intervals to provide a more accurate estimation of binomial proportions. To maintain the assumption of independence, each study contributed to one independent observation. While various quantitative synthesis approaches were considered, the high variability in data presentation and reporting format constrained the analytical methods that could be applied. 

To examine potential sources of heterogeneity, descriptive subgroup analyses were conducted considering device type (ECG-based versus PPG-based), recording duration (short-term ≤ 10 min versus long-term ≥ 24 h), and the gender composition of the study populations (female-dominant versus mixed or male-dominant).

This approach follows SWiM (Synthesis Without Meta-analysis) guidelines to summarize the overall direction of effects without relying on statistical significance thresholds. A vote-counting approach was used to determine the association pattern between HRV parameters (SDNN or RMSSD) and traditional inflammatory markers. All statistical analysis was done using R version 4.4.2.

## 3. Results

### 3.1. Search Results

Preliminary database searches yielded a total of 1035 records, comprising 346 from Scopus, 183 from PubMed, 327 from Web of Science, and 179 from the Cochrane Library. After the removal of duplicates, 584 records remained. Based on title and abstract screening, 21 studies were selected for full-text assessment to determine their eligibility for inclusion in the systematic review. Of these, four reports could not be obtained, and eight were excluded due to ineligible study design (n = 1), outcomes (n = 3), population (n = 2), and language other than English (n = 2).

**Figure 1:**
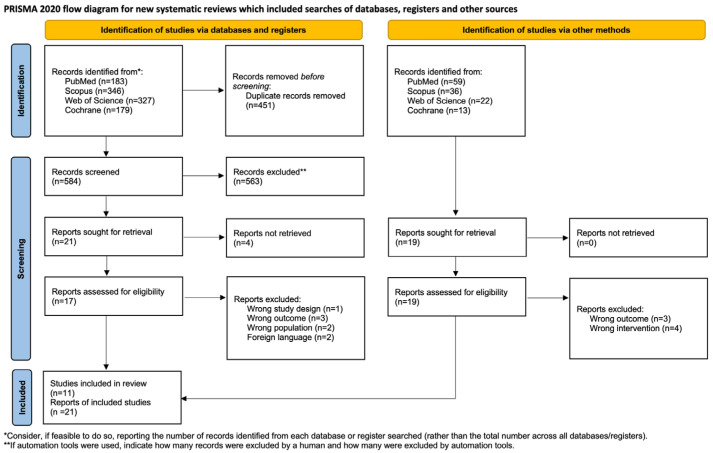
PRISMA flow diagram illustrating the study selection process for the systematic review [19].

Additionally, 130 records were identified through other sources, including manual searches and trial registries. After screening, 19 reports were assessed for eligibility, of which 7 were excluded for reasons including wrong outcome (n = 3) and wrong intervention (n = 4). In total, 11 studies met the inclusion criteria and were included in the final vote-counting review, represented across 21 reports (Figure 1).

### 3.2. Characteristics of Included Studies

Eleven studies were included in this systematic review. The total of cumulative participants were 2419 participants across various clinical contexts involving inflammatory conditions. All studies examined the relationship between HRV measured by wearable devices and systemic inflammatory biomarkers. The studies varied in study designs; 10 out of 11 studies were cohort design in nature and a single study depicted an RCT design. 

Almost all the study participants in the study had an organic inflammatory condition with the exception of Koeneman et al. which recruited healthy adult participants induced with lipopolysaccharide endotoxin to emulate inflammation. Several studies focused on specific patient populations, such as those undergoing elective colorectal surgery, experiencing severe traumatic brain injury, or presenting with COVID-19-related inflammation [20,21,22]. Others evaluated HRV in chronic inflammatory states like ulcerative colitis and preterm premature rupture of membranes (PPROMs) [13,23]. Sample sizes ranged from small cohorts of 15–20 individuals to larger groups of nearly 90 patients, with wearable monitoring conducted either continuously or at defined intervals. For example, one study included 89 patients with traumatic brain injury monitored for autonomic dysfunction, while others investigated HRV changes in 44 pregnant women and 40 surgical patients [21,23].

Regarding the wearables used in this study, the devices varied in both form and placement. Most studies employed chest-mounted wearables (6 out of 11), followed by wrist-worn devices (3 out of 11), while 1 study utilized a finger probe. The wearable technologies used included chest straps (e.g., Polar S810), biosensor patches (e.g., VitalConnect HealthPatch, Tiger Tech Warfighter Monitor), and wrist-worn devices (e.g., Ava Bracelet), each employing either photoplethysmography (PPG) or wearable ECG to derive HRV parameters. Commonly reported HRV indices were SDNN, RMSSD, LF, HF, and LF/HF ratio. A detailed overview of each study’s characteristics, including population, HRV device, biomarkers used, and key outcomes, is provided in Table 1.

#### 3.2.1. Device Recording Methods

Among the included studies, 10 out of 11 utilized electrocardiography (ECG)-based wearables, while only one study employed a photoplethysmography (PPG)-based device. The ECG-based wearables demonstrated consistent inverse associations between HRV indices (SDNN and RMSSD) and inflammatory biomarkers such as CRP, IL-6, and TNF-α [20,21,22,24,25,26,27,28,29,30]. In contrast, the single PPG-based study, which measured SDNN in relation to CRP, showed a weaker and non-significant association [23].

#### 3.2.2. Recording Duration

Among the included studies, four used long-duration recordings (>10 min) [13,23,24,25], while six applied short-term recordings (≤10 min) [20,22,27,28,30], and one study did not specify the measurement length [21]. Contrary to expectations, longer recording durations did not demonstrate stronger or more consistent associations between HRV indices (SDNN and RMSSD) and inflammatory biomarkers. Both long- and short-term recordings produced mixed effect directions, with several studies in each group showing either negative or non-significant findings.

#### 3.2.3. Chronicity of Condition

When grouped by disease chronicity, seven studies investigated chronic conditions (e.g., obesity, metabolic syndrome, epilepsy, hypertension, ulcerative colitis, and HIV infection) [22,24,25,26,27,29,30], while four studies examined acute or short-term inflammatory responses [20,21,23,28]. Chronic-condition studies generally showed negative or no associations between HRV indices (SDNN and RMSSD) and inflammatory biomarkers, particularly CRP and IL-6. In contrast, acute studies demonstrated more variable and inconsistent patterns.

### 3.3. Risk of Bias Assessment

Quality assessment using both NIH Quality Assessment l and ROB2 tools revealed moderate to low levels of bias across the included studies (Supplemental Tables S2 and S3). Overall, six studies were rated as moderate risk and four as low risk of bias across all domains from the examined observational study. The only RCT in our review was deemed as low risk of bias. Six studies were rated moderate mainly because of methodological limitations such as small sample size, limited adjustment for confounding variables, and insufficient standardization.

### 3.4. Effect Direction Plot

The outcomes of this study are presented using the effect direction plot to visualize the direction of association between RMSSD and SDNN with the inflammation biomarkers (Supplemental Tables S4 and S5). These plots provided overview of the effect of high inflammatory biomarkers level towards HRV (SDNN and RMSSD) direction. The extracted relationships between HRV and inflammatory markers include SDNN with CRP, IL-6, TNF, IL-10, Galectin-3 and IL-1ra/IL-1F3, as well as RMSSD with CRP, IL-6, IL-1, IL-10, TNF, IL-1ra/IL-1F3, fecal calprotectin, and Galectin-3, which are provided in Table 2.

#### 3.4.1. CRP

A total of 11 studies assessed the relationship between heart rate variability (HRV) and C-reactive protein (CRP), including six evaluating SDNN and five evaluating RMSSD. The study done by Haase et al. had an unclear reporting of HRV parameters in association of CRP, thus we conclude the study to be unclear for both reported HRV indices [29]. Among studies examining SDNN, five reported a negative association between HRV and CRP, while one found no association, indicating that lower SDNN values were generally associated with higher CRP levels. The proportion of studies reporting decreased HRV in the presence of heightened CRP was 0.83 with the sign test resulting in (*p* = 0.022) with a confidence interval of 0.36–1.00. Similarly, by excluding the single conflicting result, this trend was also statistically insignificant based on the sign test (*p* = 0.062), with a proportion of negative effects of 1.0 (95% CI 0.48–1.0). 

Among RMSSD studies, three of five reported a negative association, one found a positive relationship, and one showed no association. The overall trend for RMSSD suggested a weak inverse relationship that did not reach statistical significance (*p* = 0.63; 95% CI 0.19–0.99) with the proportion of 0.75 in the analysis excluding unclear data. Likewise, all studies reporting RMSSD also demonstrate an insignificant statistical result with the proportion of 0.6 (*p* = 1.00; 95% CI 0.15–0.95). A statistically significant positive association between RMSSD and CRP was observed by Hirten et al. with the report of (r =0.008 *p* <0.001); it should be noted that this association may be deemed clinically insignificant [24].

#### 3.4.2. IL-6

The association between HRV and interleukin-6 (IL-6) was reported in eight studies, three for SDNN and five for RMSSD. For SDNN, results were mixed, with two negative associations and one no association. The overall sign test indicated no significant directional consistency for all studies (proportion 0.67; *p* = 1.00; 95% CI 0.09–0.99) nor with excluding conflicting a single result (proportion 1.00; *p* = 0.5; 95% CI 0.48–1.00). The study by Barone et al. found no association between SDNN and IL-6 which may be due to the small number of samples (n = 8) reported in the study.

Among studies evaluating RMSSD, four out of five showed a negative relationship and one no association as reported by Barone et al. Statistical analysis also yielded a non-significant trend between these studies by both including all studies (proportion 0.8; *p* = 0.37; 95% CI 0.28–0.99) and excluding conflicting result (proportion 1.00; *p* = 0.12; 95% CI 0.40–1.00). Collectively, these findings suggest a tendency toward an inverse association between HRV and IL-6, although the direction and magnitude of this relationship appear to vary according to study design, recording duration, device type, and clinical context.

#### 3.4.3. TNF

A total of eight studies reported on HRV and tumor necrosis factor-alpha (TNF-α), including three assessing SDNN and five assessing RMSSD. For SDNN, two studies observed a negative association, whereas one found no relationship, producing a non-significant result for all studies (proportion 0.67; *p* = 1.00; 95% CI 0.09–0.99) and excluded conflicting results (proportion 1.0; *p* = 0.500; 95% CI 0.16–0.99). As demonstrated in the IL-6 section, Barone et al. was also reported as unclear for SNDD association with TNF. 

Among RMSSD studies, one reported a positive association, one reported a negative association, and three found an unclear association, also resulting in a non-significant pattern for all studies (proportion 0.2; *p* = 0.37; 95% CI 0.01–0.72) and excluding conflicting results (proportion 0.5; *p* = 1.0; 95% CI 0.01–0.99). Three studies, including Barone et al., were deemed unclear. Hirten et al. did not report the correlation coefficient (r) or *p*-value between RMSSD and TNF-α; on the contrary, significant associations were presented for other inflammatory biomarkers (e.g., IL-6, IL-1β, and CRP) [24]. Wang et al. likewise found no significant correlation between RMSSD and TNF-α (r = −0.045, *p* > 0.05) despite a significant reduction in TNF-α following device-guided slow breathing [25]. Hence, both studies were coded as no association in the vote-counting synthesis. Overall, TNF-α analyses revealed no consistent direction of effect, suggesting that HRV indices particularly those obtained from short wearable recordings may not reliably reflect cytokine fluctuations related to TNF-α activity.

### 3.5. Other Inflammatory Biomarkers

#### 3.5.1. IL-1

One study, by Hirten et al., evaluated the relationship between RMSSD and IL-1, reporting a negative association, in which lower RMSSD values corresponded to higher IL-1 concentrations [13]. The study reported an estimate of –0.003 (*p* = < 0.001) exhibiting a negative association between IL-1 rise and RMSSD value [24]. However, it should be noted that the risk of bias was low.

#### 3.5.2. IL-10

The association between IL-10 and HRV was examined in a single study by Koeneman et al. [28]. Both SDNN and RMSSD demonstrated a negative association with IL-10 levels with plasma IL-10 levels increased at 60 min after endotoxin administration, before a marked decrease in RMSSD and SDNN around 127–140 min. While IL-10 level increased significantly, both SDNN and RMSSD significantly decreased. We found the risk of bias to be moderate.

#### 3.5.3. Fecal Calprotectin

In one prospective cohort of ulcerative colitis patients, RMSSD demonstrated a positive association with fecal calprotectin, indicating that higher HRV values occurred in parallel with rising inflammatory activity during pre-flare phases [13]. The quality of evidence was moderate.

#### 3.5.4. IL-1F3 (IL-1ra/IL-1F3)

One study evaluated the relationship between HRV indices (SDNN and RMSSD) and interleukin-1 receptor antagonist (IL-1ra) as well as IL-1F3, and found no significant association for either parameter [29]. The quality of evidence was deemed moderate.

#### 3.5.5. Galectin-3

The galectin-3 association with SDNN and RMSSD was studied in one article [29]. Both of the HRV parameters have a negative association with galectin-3 with an association of r= −0.184 (*p* = < 0.05) for SDNN and a non-significant association of −0.042 for RMSSD. The quality of evidence was rated to be moderate.

## 4. Discussion

This systematic review synthesized available evidence on the association between wearable-derived heart rate variability (HRV) and inflammatory biomarkers. Eleven studies involving a total of 2419 participants were included. Among the HRV indices assessed, SDNN measured using wearable ECG devices appeared to show the most consistent inverse trend with C-reactive protein (CRP), whereas findings for RMSSD and inflammatory cytokines, including interleukin-6 (IL-6), tumor necrosis factor-α (TNF-α), and interleukin-10 (IL-10), were heterogeneous. Overall, these findings suggest that reduced HRV is generally associated with higher inflammatory activity, although the strength and consistency of this relationship vary by HRV metric and biomarker. Accordingly, this review should be interpreted as a structured synthesis of emerging evidence rather than a definitive evaluation, reflecting the limited number of available studies and the substantial heterogeneity in study populations, inflammatory contexts, and wearable methodologies.

Across the included studies, a total of eight inflammatory biomarkers were evaluated. Moderate-quality evidence supports an inverse association between SDNN and CRP, whereas evidence for other biomarkers remains limited, inconsistent, or derived from single studies, precluding robust inference. In contrast, biomarkers such as fecal calprotectin and galectin-3 were each represented by single studies, and their reported associations should therefore be considered exploratory. Moreover, the apparent consistency observed for SDNN-CRP should be interpreted cautiously, as sign-test results were borderline and confidence intervals were wide, reflecting limited statistical precision and the small number of contributing studies. The diversity of study populations ranging from acute inflammatory responses to chronic metabolic or cardiovascular conditions together with the predominance of observational designs restricts causal interpretation. Inconsistent reporting and adjustment for key confounders, including physical activity, medication use, circadian timing, respiratory patterns, and comorbidities, further limit generalizability across clinical contexts.

The association between inflammation and autonomic dysfunction can be explained through established neuroimmune mechanisms [31,32]. Sympathetic overactivity promotes catecholamine-mediated cytokine release, whereas reduced parasympathetic tone impairs the cholinergic anti-inflammatory reflex that normally suppresses pro-inflammatory mediators such as TNF-α, IL-1β, and IL-6 [33,34,35]. This sympathovagal imbalance leads to reductions in HRV, reflecting diminished autonomic regulation of immune activity. Acute inflammatory states, such as lipopolysaccharide exposure or trauma, may induce rapid reductions in HRV during cytokine surges, whereas chronic inflammatory conditions may sustain low HRV through persistent sympathetic dominance and vagal withdrawal [28]. These mechanistic differences suggest that acute and chronic inflammatory states may exert distinct effects on autonomic regulation. Future studies would benefit from stratifying analysis by inflammatory chronicity to better distinguish transient from sustained HRV alterations.

Evidence from cardiovascular autonomic disorders further supports the interpretation of HRV as a marker of sympathovagal balance rather than a disease-specific diagnostic signal. For example, studies in atrioventricular nodal reentrant tachycardia (AVNRT) demonstrate altered HRV patterns consistent with heightened sympathetic modulation, even in the absence of structural heart disease [36,37]. These findings underscore that HRV indices are sensitive to underlying autonomic state across diverse clinical conditions, reinforcing their role as physiological correlates of autonomic regulation rather than direct indicators of specific pathological processes.

Subgroup analyses suggested that ECG-based wearable devices yielded more consistent inverse associations between HRV indices and inflammatory biomarkers than PPG-based devices, likely due to superior signal quality and reduced susceptibility to motion artifacts [38,39,40,41]. Recording duration (>10 min versus ≤10 min) did not demonstrate a clear influence on effect direction, and both acute and chronic inflammatory cohorts exhibited variable patterns. Male-dominant or mixed-sex populations showed slightly stronger inverse associations than female-dominant populations, potentially reflecting sex-related differences in autonomic regulation. Together, these findings indicate that both methodological and physiological factors contribute substantially to heterogeneity across studies.

Throughout this review, associations between wearable-derived HRV and inflammatory biomarkers are interpreted as physiological correlations rather than evidence of diagnostic performance. Establishing such biological associations represents a prerequisite for but is conceptually distinct from the validation of HRV as a diagnostic or predictive tool. In our review, formal diagnostic metrics, such as sensitivity, specificity, and AUC, were inconsistently reported across the included studies and were often unavailable. Accordingly, a formal evaluation of diagnostic accuracy was not feasible and the translational implications of these findings are intentionally framed within an exploratory context. Thus, clinically, wearable-derived HRV may function as an investigational physiological marker but should not yet be used to guide diagnosis or management decisions.

Several limitations must be acknowledged. The SWiM-based vote-counting approach applied in this review has inherent constraints, as all included studies contribute equally regardless of sample size, precision, or methodological quality, and the synthesis does not estimate effect magnitude, diagnostic performance, or predictive value, thus this review provides a lower level of certainty regarding overall strength of evidence [18]. These methods were selected because of substantial heterogeneity primarily in outcome metrics, study design, populations, wearable technologies, and analytical approaches preventing formal quantitative meta-analysis, making a SWiM-based synthesis the most appropriate approach for summarizing qualitative patterns of association despite its inferential limitations. Beyond these analytical constraints, many included studies had small sample sizes, increasing the risk of type II error, and the predominance of observational designs limits causal inference. In addition, key confounders known to influence HRV including medication use, circadian timing, physical activity levels, respiratory patterns, and individual physiological differences were inconsistently reported or adjusted for, which may have biased observed associations. Substantial heterogeneity in study populations, wearable device technologies, recording durations, biomarker assays, and analytical protocols further complicate cross-study comparisons and may contribute to inconsistent findings across inflammatory markers. Finally, restricting inclusion to English- and Indonesian-language publications may have excluded relevant studies published in other languages, introducing potential language-related selection bias.

Overall, the available evidence indicates that SDNN derived from wearable ECG devices most consistently shows an inverse association with CRP, supporting the relevance of HRV as a non-invasive physiological correlate of systemic inflammatory activity. However, the current evidence remains preliminary. Further high-quality, standardized, and longitudinal studies are required to confirm these associations and to clarify the potential role of wearable HRV monitoring in inflammation-related research and exploratory clinical contexts.

## 5. Conclusions

Wearable-derived heart rate variability, particularly SDNN derived from wearable ECG devices, suggests a predominant inverse association with CRP relative to other HRV indices and biomarkers. However, the overall certainty of the evidence remains limited. Associations involving other inflammatory biomarkers, including interleukin-6, tumor necrosis factor-α, and interleukin-10, were heterogeneous and frequently inconsistent. Substantial variability in wearable sensor modality, recording duration, analytical approaches, and study populations further constrains interpretation and limits confidence in the observed relationships. Accordingly, current evidence supports wearable-derived HRV as an exploratory or adjunctive physiological correlate of inflammation rather than a validated diagnostic or predictive tool. Future well-designed, standardized, and longitudinal studies incorporating synchronized inflammatory reference standards and formal accuracy or prediction frameworks are required to evaluate diagnostic performance and clarify the translational potential of wearable HRV monitoring in inflammation-related research and clinical contexts.

## Figures and Tables

**Table 1:** General characteristics of studies included in present systematic review.

Study and Country	Study Design	Sample Size	Age	Female (%)	Population	HRV Measuring Device	Record Duration	HRV Parameters	Biomarkers	Key Findings [19]
(2012) Haase et al. [20] Germany	Prospective, observational (Cohort study)	40	61 (26–80)	50%	Patients undergoing elective colorectal surgery	Polar S810 Heart Rate Monitor on chest strap	10 min	SDNN, RMSSD, pNN50,LF, HF, LF/HF ratio	CRP	No correlation between change of HRV parameters and leukocyte count and CRP on postoperative day 1 or 3 (each *p* > 0.2), albeit a decreased HRV can be observed.
(2018) Deepika et al. [21] India	Prospective, longitudinal (Cohort study)	89	34.88 ± 11.06	15%	Patients with severe traumatic brain injury	Telemetric device BioHarness on chest strap (Zephyr Technologies, Annapolis, MD, USA)	NR	SDNN, RMSSD, LF/HF ratio	TNF-α, IL-6, IL-10, IL-1b	Significant correlations between HRV and cytokines: IL-6 negatively correlated with RMSSD.
(2021) Hasty et al. [22] USA	Observational (Cohort study)	16	60.5 ± 13.4	29%	COVID-19 patients admitted to ICU	Tiger Tech Warfighter Monitor on arm band	7 min	SDNN	CRP	Correlation between a >40% decrease in HRV and subsequent 50% rise in CRP.
(2021) Hirten et al. [24] USA	Prospective, observational cohort (Cohort study)	15	33 (median)	60%	Patients with ulcerative colitis	VitalPatch on chest patch (VitalConnect, San Jose, CA, USA)	72 h	RMSSD, LF, HF, LFHF	CRP, TNF, IL-6, IL-1β, Fecal calprotectin	Significant changes in HRV precede symptomatic or inflammatory flare; HRV associated with stress and UC symptoms.
(2023) Brun et al. [23] Switzerland	Prospective proof of principle (Cohort study)	44	33.8 ± 5.8	100%	Pregnant women diagnosed with preterm premature rupture of membranes (PPROMs)	Ava Bracelet on wrist band		SDNN	CRP	Significant differences in heart rate and breathing rate in women with intra-amniotic infection compared to those without.
(2023) Wang et al. [25] China	Repeated measurement observational (Cohort study)	93	23.3 ± 2.0	32%	An amount of 53 normal-weight and 44 obese young adults	12-lead ambulatory ECG on chest electrodes (MGY-H12, MEIGAOYI, China)	24 h	SDNN, rMSSD, pNN50, LF, HF, LF–HF ratio	MCP-1, IL-6, IL-8, TNF-α, fractalkine, MIP-1α, and MIP-1β	Obese individuals showed heightened susceptibility to noise effects on HRV; suggests need for tailored interventions.
(2008) Barone [26] Italia	Prospective observational study (3-month follow-up) (Cohort study)	44	32 ± 24	75%	Epilepsy patients with refractory seizures	Holter ECG on chest electrodes (Oxford Medilog Excel 3 system)	24 h	SDNN, RMSSD, pNN50, LF/HF ratio	CRP, TNF-α, IL-6	No significant changes in HRV or inflammatory markers.
(2015) Bestawros [27] South Africa	Prospective observational cohort (12-week follow-up) (Cohort study)	60	36 (IQR 31–42)	42%	A total of 33 undernourished HIV-infected adults in Zambia and Tanzania	EndoPAT on finger probe (Peripheral Arterial Tonometry HRV Sensor)	5 min	RMSSD, triangular index, power ratio, LF/HF ratio	CRP, TNF-α R1, CD163	HRV negatively correlated with inflammation; endothelial function improved as inflammation declined.
(2021) Koeneman [28] Netherlands	Randomized controlled trial (RCT), experimental human endotoxemia model (RCT)	30	22 (19–23)	NR	A total of 30 healthy volunteers (15 LPS-exposed, 15 placebo)	VitalConnect HealthPatch on chest patch (wearable single-lead ECG)	6 min	LF/HF ratio, RMSSD, SDNN	TNF-α, IL-6, IL-10	HRV changes (LF/HF rise, RMSSD decrease, and SDNN decrease) preceded symptoms and vital sign changes.
(2021) Wang [29] Taiwan	Prospective observational study (3-month intervention) (Cohort study)	36	59.4 ± 9.0	39%	Essential hypertensive patients	MiCor A100 wearable ECG on wrist band (single-lead ECG HRV sensor)	2 min	SDNN, RMSSD, pNN50, LF/HF ratio	TNF-α, IL-6, IL-1ra/IL-1F3, CRP, galectin-3	LF/HF ratio was positively correlated with TNF-α and galectin-3, while SDNN showed a negative correlation with CRP (*p* < 0.05); no significant associations for other indices.
(2024) Ochieng [30] Turkey	Comparative observational study (cross-sectional study)	27	43.83 ± 16.49	63%	An amount of 13 individuals with metabolic syndrome (MetS); 14 without MetS	Wrist-worn embedded device	6 min	RMSSD, SDNN, LF/HF ratio	CRP	Subjects with MetS showed significantly higher CRP levels and lower HRV indices (RMSSD, LF, HF, LF/HF).

CRP = C-Reactive Protein; ECG = Electrocardiogram; HF = High Frequency; LF = Low Frequency; RMSSD = Root Mean Square of Successive Differences; SDNN = Standard Deviation of Normal-to-Normal intervals.

**Table 2:** Vote counting, sign test, and proportion of negative effects.

						All Studies			Excluding Conflicting Results		
Comparison		▲	◄►	▼	Total	*p* Value	Proportion of Decreased HRV	95% CI	*p* Value	Proportion of Decreased HRV	95% CI
SDNN	CRP	0	1	5	6	0.22	0.83	0.36–1.00	0.062	1	0.48–1.00
	IL-6	0	1	2	3	1.00	0.67	0.09–0.99	0.500	1	0.48–1.00
	TNF	0	1	2	3	1.00	0.67	0.09–0.99	0.500	1.00	0.16–1.00
RMSSD	CRP	1	1	3	5	1	0.6	0.15–0.95	0.63	0.75	0.19–0.99
	IL-6	0	1	4	5	0.37	0.8	0.28–0.99	0.12	1	0.40–1.00
	TNF	1	3	1	5	0.37	0.2	0.01–0.72	1	0.5	0.01–0.99

Effect direction: ▲ = HRV indices increase in response to higher inflammatory marker levels (positive association), ▼ = HRV indices decrease in response to higher inflammatory marker levels (negative association), ◄► = No clear change, mixed, or conflicting associations between HRV and inflammatory markers. The symbols summarize the direction of reported associations and are intended to support qualitative interpretation rather than quantitative or diagnostic inference.

## Data Availability

The raw data supporting the conclusions of this article will be made available by the authors on reasonable request.

## Supplementary Materials

The following supporting information can be downloaded at: https://www.mdpi.com/article/10.3390/diagnostics16040538/s1, Table S1. Search strategy keywords for each database; Table S2. Risk of Bias Assessment (NIH Quality Assessment Tool for Observational Cohort and Cross-Sectional Studies); Table S3. Risk of Bias Assessment (RoB 2); Table S4. Effect Direction Plot for SDNN in the condition of heightened biomarker level; Table S5. Effect Direction Plot for RMSSD in the condition of heightened biomarker level; Figure S1. PRISMA Checklist.

## Abbreviations

The following abbreviations are used in this manuscript:

ACT	α1-antichymotrypsin
ANS	autonomic nervous system
CRP	C-reactive protein
ECG	electrocardiogram
HF	high frequency
HRV	heart rate variability
ICAM	intercellular adhesion molecule
IL	interleukin
LF	low frequency
MPO	myeloperoxidase
pNN50	percentage of successive NN intervals differing by more than 50 ms
PPG	photoplethysmography
PPROM	preterm premature rupture of membranes
PRISMA	preferred reporting items for systematic reviews and meta-analyses
RCT	randomized controlled trial
RMSSD	root mean square of successive differences
SDNN	standard deviation of NN intervals
SWiM	synthesis without meta-analysis
TNF-α	tumor necrosis factor alpha
UC	ulcerative colitis
VCAM	vascular cell adhesion molecule
WBC	white blood cell
