# Supplementary material for: Clinical Evidence of Wearable-Derived Heart Rate Variability for Detecting Systemic Inflammation: A Systematic Review
Source: Diagnostics (Basel). 2026 Feb 11;16(4):538. doi: 10.3390/diagnostics16040538 (PMC12939863; doi:10.3390/diagnostics16040538)
Supplement: Supplementary file 1 [file diagnostics-16-00538-s001.zip › diagnostics-4090132-supplementary.pdf]

Supplementary Material

Supplemental Table S1. Search strategy keywords for each database

Database	Keyword combination
Scopus	(TITLE-ABS-KEY("heart rate variability" OR HRV OR "autonomic function" OR ANS OR HRC OR "heart rate characteristic" OR "cardiac variability" OR "heart period variability" OR "respiratory sinus arrhythmia" OR RSA OR vagal OR vagus)<br><br>AND<br><br>TITLE-ABS-KEY("wearable" OR "device" OR "sensor" OR "smartwatch" OR "wearable technology" OR "wearable devices" OR "smart wearables" OR "smartwatches" OR "tracker*" OR "tracking" OR "step count*" OR "personal device*" OR "home monitoring" OR "mobile application" OR "remote sensing technolog*" OR "pedometer" OR "EAMDs" OR "EAMD" OR "smart-watch*" OR "actigraph*" OR "actigraphies" OR "Wii" OR "physical activity monitoring" OR "accelerometer" OR "activity tracker" OR "fitbit" OR "apple")<br><br>AND<br><br>TITLE-ABS-KEY("systemic inflammation" OR "inflammation" OR "C-reactive protein" OR "CRP" OR "cytokines" OR inflamm* OR interleuk* OR IL-6 OR IL-8 OR IL-2 OR IL-1 OR "white blood cell*" OR WBC OR leukocytes OR fibrinogen OR myeloperoxidase OR MPO OR "tumor necrosis factor" OR TNF OR "interferon gamma" OR IFNγ OR ICAM OR VCAM OR "α1-antichymotrypsin" OR ACT))
PubMed	(("Heart Rate Variability"[MeSH Terms] OR "Heart Rate Variability"[tiab] OR HRV[tiab] OR "autonomic function"[tiab] OR ANS[tiab] OR HRC[tiab] OR "Heart rate characteristic"[tiab] OR "cardiac variability"[tiab] OR "heart period variability"[tiab] OR "respiratory sinus arrhythmia"[tiab] OR RSA[tiab] OR vagal[tiab] OR vagus[tiab])<br><br>AND<br><br>("Wearable Electronic Devices"[MeSH Terms] OR "wearable"[tiab] OR "device"[tiab] OR "sensor"[tiab] OR "smartwatch"[tiab] OR "wearable technology"[tiab] OR "wearable devices"[tiab] OR "smart wearables"[tiab] OR "smartwatches"[tiab] OR "tracker*"[tiab] OR "tracking"[tiab] OR "step count*"[tiab] OR "personal device*"[tiab] OR "home

	monitoring"[tiab] OR "mobile application"[tiab] OR "remote sensing technolog*"[tiab] OR "pedometer"[tiab] OR "EAMDs"[tiab] OR "EAMD"[tiab] OR "smart-watch*"[tiab] OR "actigraph*"[tiab] OR "actigraphies"[tiab] OR "Wii"[tiab] OR "physical activity monitoring"[tiab] OR "accelerometer"[tiab] OR "activity tracker"[tiab] OR "fitbit"[tiab] OR "apple"[tiab])<br><br>AND<br><br>("Inflammation"[MeSH Terms] OR "systemic inflammation"[tiab] OR "C-reactive protein"[MeSH Terms] OR "C-reactive protein"[tiab] OR CRP[tiab] OR Cytokines[tiab] OR inflamm*[tiab] OR interleuk*[tiab] OR IL-6[tiab] OR IL-8[tiab] OR IL-2[tiab] OR IL-1[tiab] OR "white blood cell*"[tiab] OR WBC[tiab] OR leukocytes[tiab] OR fibrinogen[tiab] OR myeloperoxidase[tiab] OR MPO[tiab] OR "tumor necrosis factor"[tiab] OR TNF[tiab] OR "interferon gamma"[tiab] OR IFNy[tiab] OR ICAM[tiab] OR VCAM[tiab] OR "α1-antichymotrypsin"[tiab] OR ACT[tiab]))
Web of Science	TS=("heart rate variability" OR HRV OR "autonomic function" OR ANS OR HRC OR "heart rate characteristic" OR "cardiac variability" OR "heart period variability" OR "respiratory sinus arrhythmia" OR RSA OR vagal OR vagus)<br><br>AND<br><br>TS=("wearable" OR "device" OR "sensor" OR "smartwatch" OR "wearable technology" OR "wearable devices" OR "smart wearables" OR "smartwatches" OR "tracker*" OR "tracking" OR "step count*" OR "personal device*" OR "home monitoring" OR "mobile application" OR "remote sensing technolog*" OR "pedometer" OR "EAMDs" OR "EAMD" OR "smart-watch*" OR "actigraph*" OR "actigraphies" OR "Wii" OR "physical activity monitoring" OR "accelerometer" OR "activity tracker" OR "fitbit" OR "apple")<br><br>AND<br><br>TS=("systemic inflammation" OR "inflammation" OR "C-reactive protein" OR CRP OR cytokines OR inflamm* OR interleuk* OR IL-6 OR IL-8 OR IL-2 OR IL-1 OR "white blood cell*" OR WBC OR leukocytes OR fibrinogen OR myeloperoxidase OR MPO OR "tumor necrosis factor" OR TNF OR "interferon gamma" OR IFNy OR ICAM OR VCAM OR "α1-antichymotrypsin" OR ACT)

Cochrane Library	#1 (("Heart Rate Variability":ti,ab OR HRV:ti,ab OR "autonomic function":ti,ab OR ANS:ti,ab OR HRC:ti,ab OR "Heart rate characteristic":ti,ab OR "cardiac variability":ti,ab OR "heart period variability":ti,ab OR "respiratory sinus arrhythmia":ti,ab OR RSA:ti,ab OR vagal:ti,ab OR vagus:ti,ab)<br><br>#2 (Wearable:ti,ab OR device:ti,ab OR sensor:ti,ab OR smartwatch:ti,ab OR "wearable technology":ti,ab OR "wearable devices":ti,ab OR "smart wearables":ti,ab OR smartwatches:ti,ab OR (wearable:ti,ab OR tracker:ti,ab OR tracking:ti,ab OR ("step" NEXT count):ti,ab OR ("personal" NEXT device):ti,ab OR "home monitoring":ti,ab OR "mobile application":ti,ab OR ("remote sensing" NEXT technology):ti,ab OR pedometer:ti,ab OR EAMDs:ti,ab OR EAMD:ti,ab OR smart-watch:ti,ab OR smartwatch:ti,ab OR actigraph*:ti,ab OR actigraphies:ti,ab OR Wii:ti,ab OR "physical activity monitoring":ti,ab OR accelerometer:ti,ab OR "activity tracker":ti,ab OR fitbit:ti,ab OR apple:ti,ab))<br><br>#3 ([mh Inflammation] OR "Systemic inflammation":ti,ab OR "C-reactive protein":ti,ab OR Cytokines:ti,ab OR CRP:ti,ab OR TNF-α:ti,ab OR inflammation :ti,ab OR interleukin:ti,ab OR IL-6:ti,ab OR IL-8:ti,ab OR IL-2:ti,ab OR IL-1:ti,ab OR "C reactive protein":ti,ab OR ("white blood" NEXT cell*):ti,ab OR WBC:ti,ab OR leukocytes:ti,ab OR fibrinogen:ti,ab OR myeloperoxidase:ti,ab OR MPO:ti,ab OR "tumor necrosis factor":ti,ab OR TNF:ti,ab OR "interferon gamma":ti,ab OR IFNy:ti,ab OR ICAM:ti,ab OR VCAM:ti,ab OR α1-antichymotrypsin:ti,ab OR ACT:ti,ab))<br><br>#4 #1 AND #2 AND #3

Supplemental Table S2. Risk of Bias Assesment (NIH Quality Assessment Tool for Observational Cohort and Cross-Sectional Studies)

No.	Criteria	(2012) Haase et al	(2018) Deepika et al	(2021) Hasty et al	(2021) Hirten et al	(2022) Brun et al	(2023) Wang et al	(2008) Barone	(2015) Bestawros	(2021) Wang	(2024) Ochieng
1	Was the research question or objective in this paper clearly stated?	Yes	Yes	Yes	Yes	Yes	Yes	Yes	Yes	Yes	Yes
2	Was the study population clearly specified and defined?	Yes	Yes	Yes	Yes	Yes	Yes	Yes	Yes	Yes	Yes
3	Was the participation rate of eligible persons at least 50%?	Yes	Yes	Yes	Yes	Yes	Yes	Yes	Yes	Yes	Yes
4	Were all the subjects selected or recruited from the same or similar populations (including the same time period)? Were inclusion and exclusion criteria for being in the study prespecified and applied uniformly to all participants?	No	No	No	No	No	No	No	No	No	No
5	Was a sample size justification, power description, or variance and effect estimates provided?	No	No	No	No	No	Yes	No	No	No	No
6	For the analyses in this paper, were the exposure(s) of interest measured prior to the outcome(s) being measured?	Yes	Yes	NA	Yes	Yes	Yes	Yes	Yes	Yes	Yes
7	Was the timeframe sufficient so that one could reasonably expect to see an association between exposure and outcome if it existed?	Yes	Yes	Yes	Yes	Yes	Yes	Yes	Yes	Yes	Yes
8	For exposures that can vary in amount or level, did the study examine different levels of the exposure as related to the outcome (e.g., categories of exposure, or exposure measured as continuous variable)?	No	Yes	No	Yes	No	No	No	Yes	No	No
9	. Were the exposure measures (independent variables) clearly defined, valid, reliable, and implemented consistently across all study participants?	No	Yes	Yes	Yes	Yes	No	Yes	Yes	Yes	Yes
10	Was the exposure(s) assessed more than once over time?	No	Yes	Yes	Yes	Yes	Yes	NA	No	Yes	Yes
11	Were the outcome measures (dependent variables) clearly defined, valid, reliable, and implemented consistently across all study participants?	Yes	Yes	No	Yes	Yes	Yes	Yes	Yes	Yes	Yes
12	Were the outcome assessors blinded to the exposure status of participants?	NA	NA	NA	NA	NA	NA	NA	Yes	NA	NA
13	Was loss to follow-up after baseline 20% or less?	Yes	Yes	Yes	Yes	Yes	Yes	No	Yes	Yes	Yes
14	Were key potential confounding variables measured and adjusted statistically for their impact on the relationship between exposure(s) and outcome(s)?	NA	Yes	NA	Yes	NA	Yes	NA	Yes	No	Yes
**Total : Add scores for each criterion together and divide by 12. Risk of bias rating (Low (75-100%), Moderate (25-75%), or High (0**		50	78.57142857	50	78.57142857	64.28571429	71.42857143	50	78.57142857	64.28571429	71.42857143
**Result**		Moderate	Low	Moderate	Low	Moderate	Moderate	Moderate	Low	Moderate	Low

Supplemental Table S3. Risk of Bias Assesment (RoB 2)

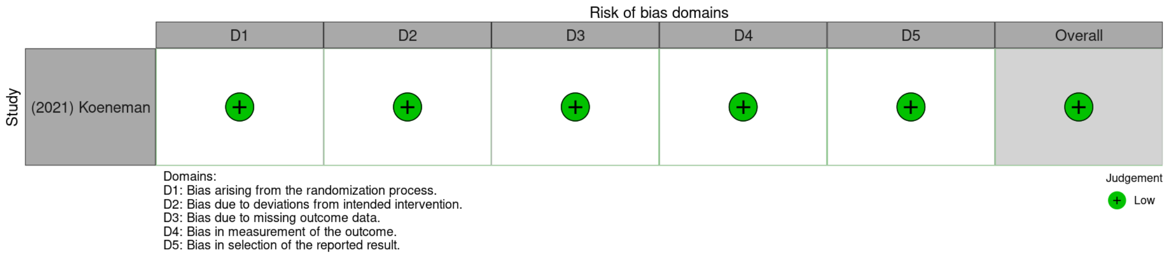

Supplemental Table S4. Effect Direction Plot for SDNN in the condition of heightened biomarker level

Author, Year	Type of study	CRP	IL-6	TNF	IL-10	Galectin-3	IL-1ra/IL-1F3	Risk of Bias
(2012) Haase et al	Cohort study	◄►						
(2018) Deepika et al	Cohort study							
(2021) Hasty et al	Cohort study	▼						
(2021) Hirten et al	Cohort study							
(2022) Brun et al	Cohort study	▼						
(2023) Wang et al	Cohort study	▼						
(2008) Barone	Cohort study		◄►	◄►				
(2015) Bestawros	Cohort study							
(2021) Koeneman	RCT		▼	▼	▼			

(2021) Wang	Cohort study	▼	▼	▼		▼	◄►	
(2024) Ochieng	Cohort study	▼						

Effect direction: ▲ = HRV indices increase in response to higher inflammatory marker levels (positive association), ▼ = HRV indices decrease in response to higher inflammatory marker levels (negative association), ◄► = No clear change, mixed, or conflicting associations between HRV and inflammatory markers

Supplemental Table S5. Effect Direction Plot for RMSSD in the condition of heightened biomarker level

**Author, Year**	**Type of study**	**CRP**	**IL-6**	**IL-1**	**IL-10**	**TNF**	**Fecal Calprotein**	**IL-1ra/IL-1F3**	**Galectin-3**	**Risk of Bias**
(2012) Haase et al	Cohort study	◄►								
(2018) Deepika et al	Cohort study		▼							
(2021) Hasty et al	Cohort study									

(2021) Hirten et al	Cohort study	▲	▼	▼		◄►	▲			
(2022) Brun et al	Cohort study									
(2023) Wang et al	Cohort study									
(2008) Barone	Cohort study		◄►			◄►				
(2015) Bestawros	Cohort study	▼				▲				
(2021) Koeneman	RCT		▼		▼	▼				
(2021) Wang	Cohort study	▼	▼			◄►		◄►	▼	
(2024) Ochieng	Cohort study	▼								

Effect direction: ▲ = HRV indices increase in response to higher inflammatory marker levels (positive association), ▼ = HRV indices decrease in response to higher inflammatory marker levels (negative association), ◄► = No clear change, mixed, or conflicting associations between HRV and inflammatory markers

Supplemental Figure S1. PRISMA Checklist

Section and Topic	Item #	Checklist item	Location where item is reported
**TITLE**			
Title	1	Identify the report as a systematic review.	Title page
**ABSTRACT**			
Abstract	2	See the PRISMA 2020 for Abstracts checklist.	Structured abstract
**INTRODUCTION**			
Rationale	3	Describe the rationale for the review in the context of existing knowledge.	Introduction, paragraph 1 - 2
Objectives	4	Provide an explicit statement of the objective(s) or question(s) the review addresses.	Introduction, final paragraph
**METHODS**			
Eligibility criteria	5	Specify the inclusion and exclusion criteria for the review and how studies were grouped for the syntheses.	Methods - Eligibility Criteria
Information sources	6	Specify all databases, registers, websites, organisations, reference lists and other sources searched or consulted to identify studies. Specify the date when each source was last searched or consulted.	Methods - Study Searching
Search strategy	7	Present the full search strategies for all databases, registers and websites, including any filters and limits used.	Methods - Table 1, full search strings in Multimetida Appendix 1

Selection process	8	Specify the methods used to decide whether a study met the inclusion criteria of the review, including how many reviewers screened each record and each report retrieved, whether they worked independently, and if applicable, details of automation tools used in the process.	Methods - Literature searching
Data collection process	9	Specify the methods used to collect data from reports, including how many reviewers collected data from each report, whether they worked independently, any processes for obtaining or confirming data from study investigators, and if applicable, details of automation tools used in the process.	Methods - Data Management & Extraction
Data items	10a	List and define all outcomes for which data were sought. Specify whether all results that were compatible with each outcome domain in each study were sought (e.g. for all measures, time points, analyses), and if not, the methods used to decide which results to collect.	Methods - Types of Outcome Measures
	10b	List and define all other variables for which data were sought (e.g. participant and intervention characteristics, funding sources). Describe any assumptions made about any missing or unclear information.	Methods - Data EXtraction & Subject Characteristics
Study risk of bias assessment	11	Specify the methods used to assess risk of bias in the included studies, including details of the tool(s) used, how many reviewers assessed each study and whether they worked independently, and if applicable, details of automation tools used in the process.	Methods - Quality ASssesment (QUADAS-2)
Effect measures	12	Specify for each outcome the effect measure(s) (e.g. risk ratio, mean difference) used in the synthesis or presentation of results.	Methods - Data Synthesis and Analysis
Synthesis methods	13a	Describe the processes used to decide which studies were eligible for each synthesis (e.g. tabulating the study intervention characteristics and comparing against the planned groups for each synthesis (item #5)).	Methods - Synthesis methods, vote counting
	13b	Describe any methods required to prepare the data for presentation or synthesis, such as handling of missing summary statistics, or data conversions.	Methods - Vote counting and sign test calculations
	13c	Describe any methods used to tabulate or visually display results of individual studies and syntheses.	Results - Tables and Effect direction plot

	13d	Describe any methods used to synthesize results and provide a rationale for the choice(s). If meta-analysis was performed, describe the model(s), method(s) to identify the presence and extent of statistical heterogeneity, and software package(s) used.	Methods - SWiM guideline, R software
	13e	Describe any methods used to explore possible causes of heterogeneity among study results (e.g. subgroup analysis, meta-regression).	Not performed
	13f	Describe any sensitivity analyses conducted to assess robustness of the synthesized results.	Not performed
Reporting bias assessment	14	Describe any methods used to assess risk of bias due to missing results in a synthesis (arising from reporting biases).	Discussion - Potential publication bias
Certainty assessment	15	Describe any methods used to assess certainty (or confidence) in the body of evidence for an outcome.	Not performed
**RESULTS**			
Study selection	16a	Describe the results of the search and selection process, from the number of records identified in the search to the number of studies included in the review, ideally using a flow diagram.	Results - Study selection, PRISMA flow diagram
	16b	Cite studies that might appear to meet the inclusion criteria, but which were excluded, and explain why they were excluded.	Results - Study selection (reasons for exclusion)
Study characteristics	17	Cite each included study and present its characteristics.	Results - Characteristics of included studies, Table 1
Risk of bias in studies	18	Present assessments of risk of bias for each included study.	Results - Risk of Bias Assessment, Figure 2
Results of individual studies	19	For all outcomes, present, for each study: (a) summary statistics for each group (where appropriate) and (b) an effect estimate and its precision (e.g. confidence/credible interval), ideally using structured tables or plots.	Results - Table 2 and narrative synthesis

Results of syntheses	20a	For each synthesis, briefly summarise the characteristics and risk of bias among contributing studies.	Results - Characteristics and Risk of Bias Summary
	20b	Present results of all statistical syntheses conducted. If meta-analysis was done, present for each the summary estimate and its precision (e.g. confidence/credible interval) and measures of statistical heterogeneity. If comparing groups, describe the direction of the effect.	Results - Vote counting and sign test
	20c	Present results of all investigations of possible causes of heterogeneity among study results.	Not performed
	20d	Present results of all sensitivity analyses conducted to assess the robustness of the synthesized results.	Not performed
Reporting biases	21	Present assessments of risk of bias due to missing results (arising from reporting biases) for each synthesis assessed.	Discussion - Limitations and publication bias
Certainty of evidence	22	Present assessments of certainty (or confidence) in the body of evidence for each outcome assessed.	Not performed
**DISCUSSION**			
Discussion	23a	Provide a general interpretation of the results in the context of other evidence.	Discussion paragraph 1-2
	23b	Discuss any limitations of the evidence included in the review.	Discussion paragraph 3
	23c	Discuss any limitations of the review processes used.	Discussion paragraph 4
	23d	Discuss implications of the results for practice, policy, and future research.	Discussion paragraph 5-6

OTHER INFORMATION			
Registration and protocol	24a	Provide registration information for the review, including register name and registration number, or state that the review was not registered.	Methods - Protocol registration (CRD420251029821)
	24b	Indicate where the review protocol can be accessed, or state that a protocol was not prepared.	Methods - Protocol registration
	24c	Describe and explain any amendments to information provided at registration or in the protocol.	Not applicable (no amendments reported)
Support	25	Describe sources of financial or non-financial support for the review, and the role of the funders or sponsors in the review.	Funding - 'None received'
Competing interests	26	Declare any competing interests of review authors.	Conflicts of interest - 'none declared'
Availability of data, code and other materials	27	Report which of the following are publicly available and where they can be found: template data collection forms; data extracted from included studies; data used for all analyses; analytic code; any other materials used in the review.	Not yet publicly available
